# The degree of food processing can influence serum fatty acid and lipid profiles in women with severe obesity

**DOI:** 10.3389/fnut.2023.1046710

**Published:** 2023-09-15

**Authors:** Karem Lays Soares Lopes, Nayra Figueiredo, Fabiana Martins Kattah, Glaucia Carielo Lima, Emilly Santos Oliveira, Maria Aderuza Horst, Lila Missae Oyama, Ana Raimunda Dâmaso, Renata Guimarães Moreira Whitton, Valéria de Souza Abreu, Amélia Cristina Stival Duarte, Gustavo Duarte Pimentel, Flávia Campos Corgosinho

**Affiliations:** ^1^Post-Graduation Program in Nutrition and Health, Faculty of Nutrition, Federal University of Goiás – PPGNUT-FANUT-UFG, Goiânia, Goiás, Brazil; ^2^Post-Graduation Program in Health Sciences, Faculty of Medicine, Federal University of Goiás, Goiânia, Goiás, Brazil; ^3^Nutrition Physiology Laboratory, Federal University of São Paulo, São Paulo, Brazil; ^4^Post-Graduation Program in Nutrition, Paulista Medicine School, Federal University of São Paulo, São Paulo, Brazil; ^5^Department of Physiology, Institute of Biosciences, University of São Paulo, São Paulo, Brazil; ^6^Alberto Hassi Hospital, Goiânia, Goias, Brazil

**Keywords:** nova food classification, ultra-processed foods, food, processed, obesity, lipoproteins, fatty acids, omega-3

## Abstract

**Background:**

The increase in the prevalence of obesity is associated with the increase in the consumption of ultra-processed foods and may be related to the increase in the disorders involving metabolism and the transport and storage of fatty acids.

**Objective:**

To evaluate the effect of processed food consumption according to the degree of processing on the serum fatty acid levels and lipid profile of women with severe obesity.

**Methods:**

This was a cross-sectional study. Data were collected from anthropometric assessments, the food frequency questionnaire (FFQ), and blood tests for lipidogram studies and serum fatty acid measurements. The foods consumed were identified through the FFQ and classified according to the degree of processing based on the NOVA rating, and the frequencies of consumption were transformed into scores, as proposed by Fornés methodology. Data were analyzed using IBM SPSS Statistics, version 21. The significance level for the analysis was set at 5%.

**Results:**

This study included 44 women with a mean age of 40.59 years and mean body mass index of 48.61 kg/m^2^. An inverse association was observed between the consumption of unprocessed and the occurrence of hypertriglyceridemia (*p* = 0.021) and levels of triglycerides (*p* = 0.047), total cholesterol (*p* = 0.030), and very low-density lipoprotein-cholesterol (*p* = 0.039). The consumption of processed foods was positively associated with the presence of hypertriglyceridemia (*p* = 0.044) and omega 6/3 ratio (*p* = 0.001) and negatively associated with total omega 3 levels (*p* = 0.011). The consumption of processed foods was positively associated with total cholesterol (*p* = 0.041) and negatively associated with the omega 3/6 ratio (*p* = 0.001). A negative correlation was found between the average consumption of ultra-processed foods (at least once a week) and serum level of high-density lipoprotein (*p* = 0.035).

**Conclusion:**

The consumption of processed and ultra-processed foods was associated with unfavorable lipid profiles and fatty acid levels in women with severe obesity. These results emphasize the importance of promoting the consumption of unprocessed food to mitigate metabolic disorders linked to processed food intake.

## 1. Introduction

Obesity is a chronic multifactorial disease characterized by an increase in adipose tissue, which generates chronic low-grade inflammation and poses health risks (1, 2). The increasing prevalence of obesity is a global public health problem. In 2020, 988 million people in the world presented obesity and it is estimated that in 2035 there will be an increase to 1,914 million representing 24% of the population (3). The prevalence of severe obesity (BMI > 40 kg/m^2^) has also been increasing in recent years and is more prevalent among women (4). Excess body weight is the sixth risk factor for the development and aggravation of many diseases, including chronic non-communicable diseases (NCDs) (5, 6). Individuals with obesity tend to have insulin resistance, which increases lipolysis and decreases lipogenesis. The increased efflux of fatty free acids (FFA) promotes ectopic fat accumulation, increases reactive oxygen species (ROS), induces apoptosis of pancreatic cells, and inhibits the insulin receptor (IRS-1) (7, 8). Furthermore, central adiposity is associated with increased concentrations of cytokines that activate inflammatory pathways. Such mechanisms increase the risk of developing cardiovascular diseases (9–11).

Over the period 2000 to 2016, the absolute global amount of all cardiovascular diseases increased over time, having as main risk factors high blood pressure, diabetes, smoking, and unhealthy diet. Food consumption is closely connected to weight gain, mainly as a modifiable factor (12, 13). Studies have shown that, in many cases, the sources of calories in the diet can be a stronger determinant of weight gain and comorbidities than calorie quantity (14). Thus, dietary composition is a determinant of the incidence and prevalence of obesity (14).

Modern lifestyle is characterized by a decrease in the consumption of unprocessed/minimally processed foods, dietary sources of monounsaturated fatty acids (MUFA) and polyunsaturated fatty acids (PUFA), which have cardioprotective and anti-inflammatory activity. However, PUFAs need to be consumed in adequate proportions, omega 6 fatty acids in excess modulate pro-inflammatory activity (15–19). In the other hand, it has been observed an increase in the consumption of processed and ultra-processed foods, which have high caloric density and contain many sugars, sodium, saturated, and trans fats (20).

Although the association between fatty acids and metabolic disturbances is being studied extensively, there is a lack of studies on the association between the degree of food processing and levels of serum fatty acids and lipid profile, especially in women with severe obesity (21–27). Thus, the present study evaluated the association between the consumption of processed food according to the extent and purpose of processing and serum fatty acids and lipids in women with severe obesity.

## 2. Methods

### 2.1. Location and type of study

This was a cross-sectional and convenience study involving patients with severe obesity (BMI > 40 kg/m^2^) from a public hospital in Goiânia, GO. This study was approved by the Research Ethics Committee of the Federal University of Goiás (Number 3.251.178) and the Ethical Committee of Research of the Dr. Alberto Rassi State Hospital (HGG) (Number 3.392.511). All the volunteers signed a written informed consent form before participating in the study.

### 2.2. Participants

**Figure 1:**
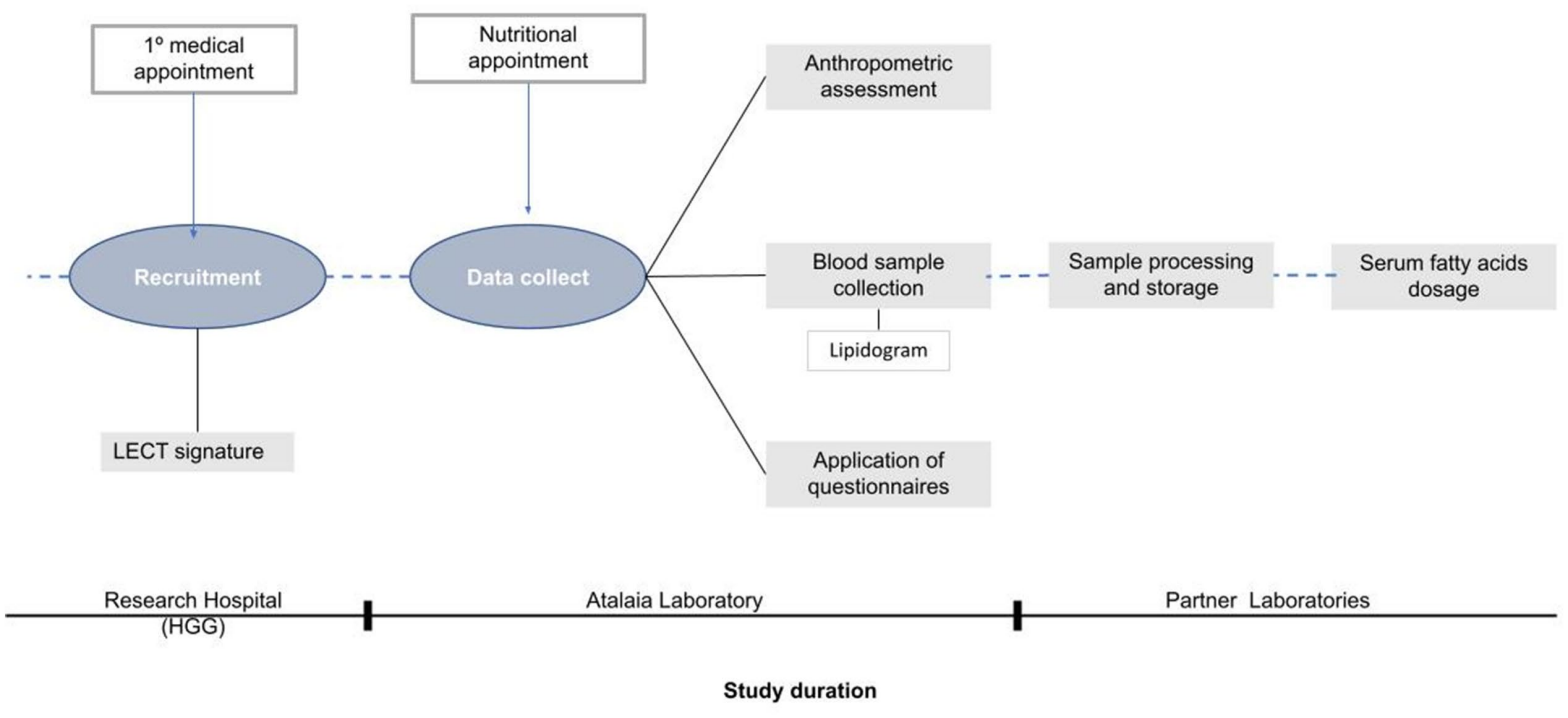
Study design: stages of participant recruitment and data collection.

The initial sample of this study was 49 participants. After an exclusion of volunteers who presented incomplete data (*n* = 3); outliers (*n* = 2), the sample became 44 volunteers aged between 20 and 59 years with severe obesity (BMI > 40 kg/m^2^) waiting for bariatric surgery at a public hospital in Goiânia were enrolled in this study. The exclusion criteria were presence of acute inflammatory diseases, infectious or neoplastic diseases, or genetic syndrome; alcohol consumption of >30 g/day; chronic use of supplements (vitamin D and omega 3) in the last 6 months; and use of drugs that cause elimination of fat via feces. All exclusion criteria were self-reported. Recruitment and data collection took place during the first consultation with the surgeon prior to any nutrition consultation and intervention (Figure 1). All our patients were recruited at the same moment, at the first appointment of the surgeon and the data was collected all at once, at the first dietitian appointment, before any dietetic guidance. Moreover, all the participants were asked if they lost weight in the last month and the majority did not change their weight previously of the data collect.

### 2.3. Data collect

Anamnesis (name, age, presence of comorbidities, medications) data were collected. Data from anthropometric assessments and blood examination were recorded, and a food frequency questionnaire (FFQ) was also applied.

#### 2.3.1. Anthropometric assessment

The measurements of weight, height, waist circumference (WC), hip circumference (HC), and neck circumference (NC) were recorded. All measurements were performed with the participants in light clothes and without shoes, and weight was measured using a weighing balance (Lider, place of manufacture), with a capacity of 200 kg (28). Height was measured using an inextensible metric, and the volunteers were asked to stand in an upright position. The body mass index (BMI) was calculated by dividing the weight by height squared. Circumferences were evaluated using an inelastic measuring tape (29) WC was measured at the level of the umbilical line while the volunteer was standing. The HC was measured at the largest circumference of the hip region (30). The NC was measured just below the level of the thyroid cartilage (31). NC and WC were evaluated and classified on cardiovascular risk and classified in low and high cardiovascular risk (≥34 and ≥88 centimeters respectively) (32, 33).

#### 2.3.2. FFQ and anamnesis

To collect information regarding the usual diet of volunteers, it used the reduced and qualitative version of the FFQ ELSA-Brasil (2013) which includes 95 foods (34). The FFQ data were tabulated in Microsoft Excel 365, and the foods listed in the questionnaire were categorized according to the degree of processing (Supplementary Chart 1), using the guidelines presented in the Food Guide for the Brazilian Population (2014), based on the NOVA rating (35, 36). Each FFQ food was classified into one of the following three groups: unprocessed/minimally processed foods, processed foods, and ultra-processed foods. The categories of culinary ingredients were not included in this study because the FFQ does not cover such items. For statistical analysis, the food consumption frequencies from the FFQ were transformed into numerical values, namely consumption scores, according to the recommendations of Fornés et al. (37). The following formula was used:


$$S_{n}=\left(1/365\right)\left[\left(a+b\right)\right]/2$$

where *a* and *b* indicate the number of days that each consumption frequency represents in the period of 1 year. For example, the consumption frequency of one-to-three times a month, *a* would be equal to 12 (considering the consumption of once a month in a period of 12 months) and *b* would be equal to 36 (considering the consumption of three times a month over a period of 12 months). For consumption frequencies of one or more times per day, the score was considered to be equal to 1 (37). The FFQ scores identified for each frequency of consumption are shown in Table 1.

**Table 1:** Food consumption frequencies calculated according to Fornés et al. (2002) (37).

Consumption frequency	Corresponding Score
3 or more times a day	1
2 or 3 times a day	1
1 time a day	1
5 or 6 times a week	0.7856
2 or 4 times a week	0.4284
1 time a week	0.1428
1 to 3 times a month	0.0657
Never or almost never	0

Subsequently, for statistical analysis, it was considered the average consumption of scores of each degree of processing. The average consumption score is related to the average frequency of consumption, referring to the number of days of the year for each degree of processing.

#### 2.3.3. Blood sample exam

For blood collection, the volunteers were instructed to fast for at least 8 h but not more than 12 h. The collection was performed by trained and qualified professionals through a peripheral puncture of a vein in the forearm.

A lipidogram examination was conducted to determine the levels of triglycerides (TGs), total cholesterol (TC), low-density lipoprotein cholesterol (LDL-c), high-density lipoprotein cholesterol (HDL-c), and very low-density lipoprotein cholesterol (VLDL-c). Biochemical analysis was performed using the colorimetric enzymatic method, specific to each parameter. Of note, the presence of different dyslipidemias was confirmed using the values of TGs, LDL-c and HDL-c, according to the laboratory classification of dyslipidemias in the Updated Brazilian Guidelines on Dyslipidemias and Prevention of Atherosclerosis (38). In addition, an aliquot of blood from each collected sample was centrifuged to separate the serum from the whole blood sample and stored in a freezer at −80°C until further testing for fatty acids.

#### 2.3.4. Extraction and determination of serum fatty acids

The fatty acid profile was determined after extracting the fatty acids from the serum samples; then, the fatty acid patterns of the samples were determined using gas chromatography. The reading and identification of the patterns of acids present in the samples were performed using chromatograms by comparison with known retention times established. The program used in the last step was Compass Chromatography Data System (Compass CDS), version 3.0.2. After the analysis results all the fatty acids from the omega-3 and omega-6 series were summed for the calculation of the ratios omega-3/omega-6 and omega-6/omega-3, an anti-inflammatory and pro-inflammatory index, respectively (17–19). All the analyses regarding the fatty acids were conducted in partnership with the Nutrition Physiology Laboratory of the Federal University of São Paulo and the University of São Paulo (39, 40).

#### 2.3.5. Statistical analysis

Statistical analysis was performed using the IBM SPSS Statistics program, version 21. The normality of the variables and residuals were evaluated using the Shapiro–Wilk test. For the analysis of the association of the different degrees of food processing was used the Generalized Linear Model test (GLzM) in exponential Family considering a normal probability of distribution (Gaussian) and a link function of identity. To investigate the association between food consumption and variables related to lipid profile (triglycerides, total cholesterol, HDL-c, LDL-c, and VLDL-c) and serum fatty acid, we performed a model of linear regression. The lipid profile was adjusted by age and BMI (main effects), since both are established influencing factors of this parameter. We also performed linear regression to investigate the association between food consumption and serum fatty acids. The homoscedasticity was tested by Levene’s test. Finally, the sample loss analysis was calculated (Supplementary Table S1).

## 3. Results

### 3.1. Sample description

A total of 44 women with an average age of 40.59 years and BMI of 48.61 kg/m^2^ were included in this study. The sample loss analysis did not show statistical difference. With regard to circumference measurements, 100% of the women had an increased risk of developing cardiovascular diseases according to the WC, and of developing metabolic disorders according to the NC (Table 2).

**Table 2:** Descriptive statistics of the lipid profile and other variables.

Variables	Mean		SD*
Age (years)	40.59	±	8.76
Weight (kg)	122.84	±	18.15
Height (m)	1.59	±	0.06
BMI (kg/m^2^)**	48.61	±	6.88
WC (cm)***	131.22	±	12.61
HC (cm)	145.64	±	13.76
NC (cm)	41.82	±	3.13
Triglycerides (mg/dL)	141.55	±	57.29
Total Cholesterol (mg/dL)	177.66	±	31.76
HDL-c (mg/dL)	47.43	±	10.39
LDL-c (mg/dL)	105.61	±	28.21
VLDL-c (mg/dL)	24.75	±	7.20
Average consumption score of unprocessed	0.23	±	0.06
Average consumption score of processed foods	0.18	±	0.08
Average consumption score of ultra-processed foods	0.10	±	0.06

SD*, standard deviation; BMI, body mass index; WC, waist circumference (<80 cm); HC, hip circumference; NC, neck circumference (≤34 cm); triglycerides (<150 mg/dL); TC, total cholesterol (<190 mg/dL); HDL-c, high-density lipoprotein (>40 mg/dL); LDL-c, low-density lipoprotein (<130 mg/dL); VLDL-c, very-low-density lipoprotein.

Regarding food consumption (Table 2), it was found that the average score for consumption of unprocessed was 0.23, indicating consumption between one and four times a week; the average scores for consumption of processed and ultra-processed foods were 0.18 and 0.10, respectively, indicating that the volunteers consumed them at least once a week. Regarding the presence of dyslipidemia, 72.7% of the volunteers had some type of alteration in the lipid profile, with the most prevalent being a reduction in HDL-c level (81.25%).

Considering the fatty acids, twenty-two were identified and included in the study. Of these, five were saturated fatty acids (SFAs), five were monounsaturated fatty acids (MUFAs), and 12 were polyunsaturated fatty acids (PUFAs) (Table 3). Furthermore, SFA showed the highest occurrence in the sample, with an of 40.72%, followed by PUFA, with 36.05%, and MUFA showed the lowest occurrence, with an average of 23.22%. The average of omega 3 and 6 fatty acids were 6.95 and 29.10%, respectively. The omega 3/6 ratio, which is an indicator of anti-inflammatory activity, had an average of 0.25%. On the other hand, the mean % of omega 6/3 ratio, a pro-inflammatory marker, was 5.16%. The fatty acid profiles are presented in Table 3.

**Table 3:** Descriptive statistics of serum fatty acid data.

Variables	Mean		SD*
**Total saturated fatty acids (% area)**	40.72	±	2.74
C14:0 (% area)	6.21	±	2.75
C16:0 (% area)	20.76	±	4.20
C18:0 (% area)	12.74	±	1.91
C20:0 (% area)	0.80	±	0.29
C22:0 (% area)	0.21	±	0.19
**Total monounsaturated fatty acids (% area)**	23.22	±	2.11
C14:1C (% area)	4.61	±	1.96
C16:1n7 (% area)	1.31	±	0.50
C18:1n9 (% area)	14.78	±	3.55
C18:1n7 (% area)	1.48	±	0.34
C20:1n9 (% area)	1.05	±	0.43
**Total polyunsaturated fatty acids (%area)**	36.05	±	3.89
C18:2n6 (% area)	19.07	±	5.35
C18:3n6 (% area)	4.86	±	1.76
C20:2n6 (% area)	2.45	±	1.04
C20:3n6 (% area)	0.36	±	1.19
C20:4n6 (% area)	0.65	±	1.27
C22:2n6 (% area)	1.71	±	0.90
**Omega 6 total (% area)**	29.10	±	3.96
C18:3n3 (% area)	3.93	±	2.08
C18:4n3 (% area)	0.72	±	0.36
C20:3n3 (% area)	1.07	±	0.96
C20:4n3 (% area)	0.40	±	0.19
C20:5n3 (% area)	0.11	±	0.42
C22:6n3 (% area)	0.71	±	0.60
**Omega 3 total (% area)**	6.95	±	3.12
**Omega 3/6 ratio**	0.25	±	0.14
**Omega 6/3 ratio**	5.16	±	2.46

SD*, standard deviation; C14:0, myristic acid; C16:0, palmitic acid; C18:0, stearic acid; C20:0, arachidic acid; C22:0, behenic acid; C:16:1n7, palmitoleic acid; C:18:1n7, vaccenic acid; C:20:1n9, cetoleic acid; C18:2n6, linoleic acid; C18:3n6, gamma-linolenic acid; C18:3n6, gamma linolenic acid; C20:2n6, eicosadienoic acid; C:20:3n6, dihomo-gamma-linolenic acid; C20:4n6, arachidonic acid; C22:2n6:13,16, docosadienoic acid; 18:3n3, alpha-linoleic acid; C18:4n3, stearidonic acid; C20:3n3, dihomo-alpha-linolenic acid; C20:4n3, eicosatetraenoic acid; C20:5n3, eicosapentaenoic acid (EPA); C22:6n3, docosahexaenoic acid (DHA).

### 3.2. Food consumption according to the degree of processing and the lipid profile

A negative correlation was observed between the variables average consumption score of ultra-processed foods and HDL-c level (*p* = 0.035). For the other variables, correlation coefficients were not significant. Regarding the relationship between the lipid profile and degree of food processing, it was found that the average consumption of unprocessed was negatively associated with the levels of TGs (*p* = 0.047; *β* = −0.371), TC (*p* = 0.030; *β* = −0.223), and VLDL-c (*p* = 0.039; *β* = −0.049), under the effect of age (Table 4).

**Table 4:** Associations between food consumption according to degree of processing and lipid profile, adjusted by age and BMI.

Dependent variable	*B*	*p*-value	95% CI
**Triglycerides**			
Score_Total_Intake_Unprocessed	4.413	0.747	(−22.347–31.173)
Score_Total_Intake_Processed	8.088	0.954	(−268.698–284.875)
Score_Total_Intake_Ultraprocessed	−5.974	0.918	(−119.961–108.013)
Score_Total_Intake_Unprocessed * Age	−0.371	**0.047**	(−0.737–−0.004)
Score_Total_Intake_Unprocessed * BMI	0.275	0.206	(−0.151–0.701)
Score_Total_Intake_Processed * Age	1.972	0.221	(−1.188–5.132)
Score_Total_Intake_Processed * BMI	−1.348	0.558	(−5.858–3.163)
Score_Total_Intake_Ultraprocessed * Age	0.719	0.270	(−0.559–1.996)
Score_Total_Intake_Ultraprocessed * BMI	−0.503	0.609	(−2.430–1.425)
**Total Cholesterol**			
Score_Total_Intake_Unprocessed	10.301	0.170	(−4.405–25.007)
Score_Total_Intake_Processed	−52.822	0.496	(−204.932–99.287)
Score_Total_Intake_Ultraprocessed	−41.567	0.193	(−104.209–21.075)
Score_Total_Intake_Unprocessed * Age	−0.223	**0.030**	(−0.424–−0.021)
Score_Total_Intake_Unprocessed * BMI	−0.027	0.821	(−0.261–0.207)
Score_Total_Intake_Processed * Age	1.815	0.041	(0.078–3.551)
Score_Total_Intake_Processed * BMI	−0.496	0.695	(−2.975–1.982)
Score_Total_Intake_Ultraprocessed * Age	0.468	0.191	(−0.234–1.170)
Score_Total_Intake_Ultraprocessed * BMI	0.414	0.444	(−0.645–1.473)
**Very-low-density lipoprotein**			
Score_Total_Intake_Unprocessed	0.626	0.720	(−2.791–4.043)
Score_Total_Intake_Processed	−0.367	0.984	(−35.709–34.975
Score_Total_Intake_Ultraprocessed	−2.059	0.782	(−16.614–12.495)
Score_Total_Intake_Unprocessed * Age	−0.049	**0.039**	(−0.096–−0.002)
Score_Total_Intake_Unprocessed * BMI	0.032	0.250	(−0.022–0.086)
Score_Total_Intake_Processed * Age	0.296	0.150	(−0.107–0.699)
Score_Total_Intake_Processed * BMI	−0.188	0.522	(−0.764–0.388)
Score_Total_Intake_Ultraprocessed * Age	0.096	0.248	(−0.067–0.259)
Score_Total_Intake_Ultraprocessed * BMI	−0.041	0.741	(−0.288–0.205)

BMI, body mass index. Model of linear regression.

On the other hand, the average consumption of processed foods was positively associated with serum TC levels, and this association was modified by advancing age (*p* = 0.041; *β* = 1.815). In addition, the average consumption of unprocessed was negatively associated (*p* = 0.021; *β* = −0.083) and the average consumption of processed foods was positively associated (*p* = 0.044; *β* = 0.335) with the presence of hypertriglyceridemia, with the interaction of age.

### 3.3. Food consumption according to the degree of processing and the profile of serum fatty acids

The mean consumption of processed foods was negatively associated with serum levels of total omega 3 (*p* = 0.008; *β* = −12.64). In the analysis of the ratios of PUFA families, we found a negative association between the average consumption of processed foods and the omega 3/6 ratio (*p* = 0.001; *β* = −1.094) and a positive association between the average consumption of processed foods and omega 6/3 ratio (*p* = 0.001; *β* = 18.751) (Table 5). No significant results were observed for the Total SFA, Total PUFA and Total MUFA variables.

**Table 5:** Associations between food consumption according to degree of processing serum fatty acids.

Dependent variable	*B*	*p*-value	95% CI
**Total Omega 3**			
Score_Total_Intake_Unprocessed	0.178	0.698	(−0.720–1.076)
Score_Total_Intake_Processed	−12.644	**0.008**	(−21.932–−3.356)
Score_Total_Intake_Ultraprocessed	2.018	0.301	(−1.808–5.843)
**Omega 3/6 Ratio**			
Score_Total_Intake_Unprocessed	0.021	0.501	(−0.040–0.082)
Score_Total_Intake_Processed	−1.094	**0.001**	(−1.729–−0.459)
Score_Total_Intake_Ultraprocessed	0.243	0.068	(−0.018–0.505)
**Omega 6/3 Ratio**			
Score_Total_Intake_Unprocessed	−0.205	0.711	(−1.289–0.879)
Score_Total_Intake_Processed	18.751	**0.001**	(7.536–29.966)
Score_Total_Intake_Ultraprocessed	−3.877	0.100	(−8.496–0.741)

Model of linear regression.

## 4. Discussion

The unquestionable role of diet both in the onset of obesity and in the development of related diseases, such as cardiovascular disease, makes it essential to thoroughly investigate food consumption and its metabolic effects in individuals who present obesity. The classification of foods according to the degree of processing has been used worldwide as an attempt to promote healthy eating and curb the growth of NCDs (41). In this sense, the present study proves to be important for the construction of knowledge about the theme of severe obesity and food intake, according to their processing levels, and fatty acids. To our knowledge, this study is the first to observe a potentially deleterious association between the consumption of processed and ultra-processed foods and levels of serum PUFAs in women with severe obesity.

The present study demonstrated an important association between processed food consumption and omega-3 fatty acids and the pro-inflammatory n6/n3 ratio. Supporting this finding, (42) observed that individuals who consumed a healthy diet (rich in whole grains, fatty fish, and “berries”) showed an increase in omega 3 series PUFAs and decline in the amounts of omega 6 and 7 fatty acids, when compared to the control group that consumed a diet rich in refined flours and sugar, with limited consumption of fish and no berries. In addition, the control group showed a reduction in the total concentration of serum omega 3 and an apparent reduction in the total concentration of circulating PUFAs from the beginning to the end of the intervention (43).

**Figure 2:**
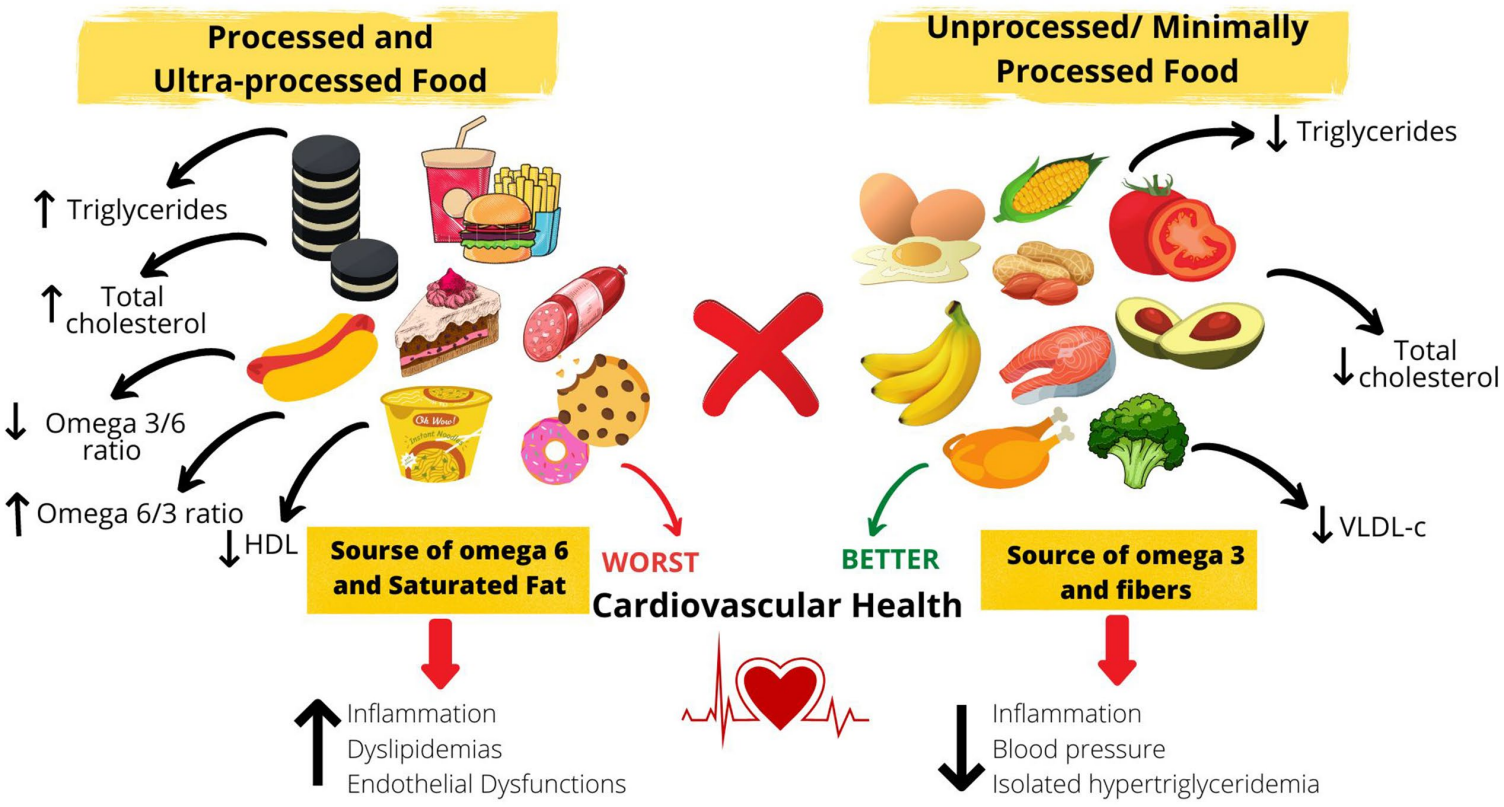
Impact of consumption of processed food, according to degree of processing, on serum fatty acids and the consequences.

The present findings could be partially explained by the food sources of omega 3 and 6. Omega 3 PUFAs are commonly found in unprocessed foods, with little or no occurrence in processed and ultra-processed foods (43). Series 6 PUFAs are abundant in oils of vegetable origin, which are frequent components of industrially produced or processed foods. In addition, the consumption of unprocessed/minimally processed, processed, and ultra-processed foods can have different effects on endogenous metabolism and may alter the metabolism of fatty acids in the body in different ways. Therefore, the consumption of unprocessed/minimally processed and industrialized foods could have influenced the amount of PUFAs found in the serum of the study volunteers (Figure 2).

Considering the roles of these essential fatty acids in the body, inadequate intake can be harmful to health (44). Omega-3 fatty acids are often associated with the reduction of systemic inflammation in overweight individuals because of their action in the production of anti-inflammatory cytokines and against the occurrence of cardiovascular events (45). Thus, the negative association between processed food and serum n-3 PUFAs is an important data, especially considering the studied population, which probably presents an increased systemic pro-inflammatory profile.

Changes in the world food pattern have favored a higher consumption of omega-6 fatty acids, which are mainly present in processed and ultra-processed foods. Omega 6, in higher proportions, can have deleterious effects on health due to its ability to produce bioactive lipids that act as pro-inflammatory agents (46). In the fatty acid biosynthesis pathway, those of the omega 6 series, mainly linoleic fatty acid, are bioconverted through the action of desaturase and elongase enzymes, giving rise to other fatty acids. Among them, arachidonic acid (ARA) stands out, which has a high inflammatory action (47). ARA can be bio converted into bioactive lipids (eicosanoids) called prostaglandins, thromboxanes, leukotrienes, and lipoxins. Eicosanoids increase vasoconstriction, bronchoconstriction, and activate inflammation by increasing the production of reactive oxygen species (ROS), promoting NK cell recruitment, and mediating the production of pro-inflammatory cytokines. Together, exposure to these metabolic changes favors the development of NCDs (48–51).

Studies have shown relevant results pertaining to the proportions of omega-6 and 3 PUFAs, where omega 6/3 ratios of 4–6:1 were associated with reduced cardiovascular risk and inflammatory parameters (44, 52, 53). On the other hand, a high omega 6/3 ratio (20:1) was related to the emergence of dyslipidemia, endothelial dysfunction, and increased inflammation (54). In experimental studies, a low omega 6/3 ratio (4:1) was found to reduce the risk of cardiovascular diseases, by reducing oxidative stress, improving endothelial function, and reducing the occurrence of inflammatory parameters. Hence, the results of the present data are alarming, once it was observed an association between consumption of processed food and omega 6/3 ratio and the mean average of consumption of processed food (once a week) in the present population was above the recommendations.

In contrast, the omega 3/6 ratio seems to have anti-inflammatory and cardiovascular protection activity. A study showed that high omega 3/6 ratio (1:1) was associated with the prevention of obesity and insulin resistance by suppressing TLR-4 (55). Additionally, it was demonstrated that n-3 PUFAs reduce the formation of atherogenic plaques by inhibiting the activation of smooth muscle cells and macrophages and help in the regulation of lipoproteins and TGs, thereby reducing the synthesis of LDL-c and TGs in the liver (56). Hence, the inverse association between ultra-processed food and serum n3/n6 in women with severe obesity highlights the importance of nutritional education actions in order to favor cardiovascular health.

In fact, the present study reinforces the importance of food processing in lipid profile. We have demonstrated that the consumption of ultra-processed food once a week was associated with a worse lipid profile while the consumption of unprocessed food was associated with a lower prevalence of hypertriglyceridemia and best lipid profile in women with severe obesity. In a study performed with adults (men and women) with hypertension, Ferreira et al. (2019) found a positive correlation between the consumption of processed foods and high TC levels (56). In line with these results, Tavares et al. (2012) and Steele et al. (2019) observed a strong correlation between the consumption of ultra-processed foods and the occurrence of metabolic syndrome; one of the components of this syndrome is the presence of HDL-c below the recommendations, which was the most common one in the study of Tavares et al. (57, 58). Moreover, Sofi et al. (2018) observed that vegetarian and Mediterranean diets were able to reduce the levels of TG, TC, and LDL-c in individuals after 3 months of the intervention (59). Together, the findings indicate a possibly beneficial effect of the consumption of foods with a low degree of processing on metabolic parameters, whereas increased consumption of processed food may be associated with the development of chronic non-communicable diseases.

It is already well-established in the literature that the consumption of foods rich in fiber can reduce the risk of hypertriglyceridemia (60). Dietary fibers increase the excretion of cholesterol-rich bile salts and reduce fat absorption by the intestine (61). Thus, the cardioprotective factor of the consumption of unprocessed food may partially explain the negative association with hypertriglyceridemia as well the high prevalence of dyslipidemia in women in the present study, since the average consumption of unprocessed food was between 1–4 times a week while processed and ultra-processed foods were at least once a week, contradicting the guidelines of the Food Guide for the Brazilian Population (36).

On the other hand, the high consumption of saturated and trans-fat, sugar, and additives presented in processed and ultra-processed food might favor dyslipidemia, inflammation, and cardiovascular disease. Saturated fatty acids are associated with worse metabolic outcomes due to their ability to impair hepatic glucose and lipid metabolism, favoring atherosclerosis and hypertriglyceridemia (62) they also promote insulin resistance (63), stress oxidative, and activate inflammatory signaling, through the Toll-like receptor (TLR) (64). Meanwhile, the excess of sugar in the diet is converted and oxidized to form acetyl-coenzyme A, a molecule necessary for the synthesis of triglycerides via lipogenesis again, favoring lipid imbalance (65). In addition, excessive sugar consumption leads to hyperglycemia, which in turn activates the *NF-KappaB* transcription factor, activating inflammatory pathways (66, 67).

In summary, the results highlight the importance of the degree of processing food in the fatty acids and lipid profile (Figure 2), emphasizing the effort of public health strategy to improve diet quality among the population, specially in patients with severe obesity, where the cardiometabolic risk is already increased (9). It is important to note that other factors, not evaluated in this study, might influence fatty acids and lipid profile, such as sleep hours and quality (68), sedentary behavior, tabagism, and presence of other diseases and genetics; and should be investigated in future studies (69, 70).

One of the limitations of the present work was the convenience study. Also, the cross sectional study does not allow causality conclusions, thus, the present results must be confirmed in longitudinal and populational study. A larger sample size could have produced stronger correlations between the variables. However, it is important to highlight that despite the sample size, several associations were found between the variables examined in this study; the sample power showed 0.80. Furthermore, because of the absence of consumption frequency data for specific foods, we could not identify specific foods that could contribute to the associations found in this study.

On the other hand, the present study is a pioneer in investigating the effect of the consumption of foods categorized using a recent classification established according to the degree of processing on the fatty acid profile, which is a metabolic parameter that is still not widely explored. Furthermore, this work can be considered relevant since it studied a population of women who belonged to a category of nutritional status that has not been examined extensively, despite its importance.

## 5. Conclusion

The present study observed negative associations between the consumption of processed and ultra-processed food with unfavorable lipid profiles and fatty acid levels in women with severe obesity, whereas unprocessed/minimally processed food favors lipid profile. It is the role of nutrition to reinforce that processed and ultra-processed foods should be avoided not only because of the caloric excess they offer, but also because of the nutritional quality that can have deleterious effects on health. These results emphasize the importance of understanding the effect of food consumption in Depper biomarkers of the metabolism and how this can impact health and disease, showing the need for further studies in different populations.

## Data availability statement

The original contributions presented in the study are included in the article/Supplementary material, further inquiries can be directed to the corresponding authors.

## Ethics statement

The studies involving humans were approved by Research Ethics Committee of the Federal University of Goiás (Number 3.251.178) and the Ethical Committee of Research of the Dr. Alberto Rassi State Hospital (HGG) (Number 3.392.511). The studies were conducted in accordance with the local legislation and institutional requirements. The participants provided their written informed consent to participate in this study.

## Author contributions

KL and FC: responsible for the writing of the original draft. KL, NF, FK, EO, GP, and FC: reviewing and editing the manuscript. KL, LO, ADâ, RW, GP, and FC: acquisition, analysis, or interpretation of data. KL, GL, MH, VS, ADu, GP, and FC: conceptualization of the study, statistical analysis, and accessed the database and raw data. FC: supervision. All authors contributed to the article and approved the submitted version.

## Funding

This research was funded by “Conselho Nacional de Desenvolvimento Científico e Tecnológico” (CnPq) grant number [no. 434159/2018-2]” and “Fundação de Apoio à Pesquisa” (FUNAPE-UFG) [no. 01/2022].

## Conflict of interest

The authors declare that the research was conducted in the absence of any commercial or financial relationships that could be construed as a potential conflict of interest.

## Publisher’s note

All claims expressed in this article are solely those of the authors and do not necessarily represent those of their affiliated organizations, or those of the publisher, the editors and the reviewers. Any product that may be evaluated in this article, or claim that may be made by its manufacturer, is not guaranteed or endorsed by the publisher.
