# Supplementary material for: The degree of food processing can influence serum fatty acid and lipid profiles in women with severe obesity
Source: Front Nutr. 2023 Sep 15;10:1046710. doi: 10.3389/fnut.2023.1046710 (PMC10541961; doi:10.3389/fnut.2023.1046710)
Supplement: Supplementary file 1 [file Data_Sheet_1.docx]

Supplementary data

Chart 1. Food items assessed by Food Frequency Questionnaire (FFQ) and classifications based on degree of processing

| Unprocessed/ minimally processed | Brown rice |
| --- | --- |
|  | White rice |
|  | oats/granola/bran/other cereals |
|  | Farofa/salted couscous/couscous from São Paulo |
|  | Cassava flour/corn flour |
|  | Cooked corn meal/angu/pirão |
|  | Boiled potato/soaked potato/mashed potatoes |
|  | Cassava/ yam / boiled banana / boiled sweet potato |
|  | Orange/tangerine/tangerine pokan (bergamot) |
|  | Banana |
|  | Papaya |
|  | Apple/ Pear |
|  | Watermelon |
|  | Melon |
|  | Pineapple |
|  | Mango |
|  | Grape |
|  | Lettuce |
|  | Sautéed kale/spinach |
|  | Cabbage |
|  | Chicory / watercress / arugula / raw kale / chicory / endive / raw chard / raw Spinach |
|  | Tomato |
|  | Pumpkin |
|  | Zucchini (Italian)/chayote/eggplant |
|  | Pod |
|  | Okra |
|  | Carrot |
|  | Beet |
|  | Cauliflower |
|  | Broccoli |
|  | Corn |
|  | Beans (black, red, white, string beans, etc.). |
|  | Lentil/chickpeas/peas |
|  | Walnuts/cashews/Brazil nuts/peanuts/almonds/pistachios |
|  | Boiled egg |
|  | Poached egg |
|  | Fried egg |
|  | Scrambled egg |
|  | Omelet |
|  | Skimmed milk |
|  | Semi-skimmed milk |
|  | Milk |
|  | Tripe |
|  | Boneless beef (steak, ground beef, stew) |
|  | Pork |
|  | Chicken breast/chester/turkey/etc. |
|  | Cooked chicken (other parts) |
|  | Cooked fish (moqueca capixaba)/roasted/stewed/grilled fish |
|  | Fried fish |
|  | Pasta (cannelloni, lasagna, ravioli, tortei) |
|  | Shrimp bean ball |
|  | legume soup |
|  | Coffee with sugar |
|  | Coffee without sugar |
|  | Natural juice with sugar |
|  | Natural juice without sugar |
|  | Chimarrão |
| Processed food | French bread/shaped/syrian/toasted |
|  | Sweet bread/homemade bread |
|  | Cheese bread |
|  | White cheeses (minas/frescal/ricotta/cottage/buffalo mozzarella) |
|  | Yellow cheeses (standard mines, mozzarella, plate, cheddar, processed canasta, etc.) |
|  | Beer |
|  | Red wine |
|  | Butter |
|  | White wine |
| Ultra-processed food | Light white bread |
|  | Whole wheat light bread |
|  | Wholemeal/rye bread |
|  | Plain cake (no filling) |
|  | Crackers (water and salt type and others) |
|  | Sweet biscuit with filling |
|  | Sweet biscuit without filling |
|  | Soy milk |
|  | Light yogurt |
|  | Regular yogurt |
|  | Margarine/vegetable cream |
|  | Sausage/chorizo ​​ |
|  | Ham/mortadella/copa/salami/pate/etc. |
|  | Pizza |
|  | Baked snacks (esfirra, pie, empanada/oven pastry, etc.) |
|  | Creamy ice cream |
|  | Bar chocolate/bonbon/brigadeiro (dark), dulce de leche, party candy |
|  | pudding/milk-based jam/mousse |
|  | Diet/light soda |
|  | Normal soda |
|  | Coffee with sweetener |
|  | Natural juice with sweetener |
|  | Industrialized juice with sugar |
|  | Unsweetened processed juice |
|  | Industrialized juice with teenager |
|  | Artificial juice with sugar |
|  | Artificial juice without sugar |
|  | Aritificial juice with sweetener |
|  | Distilled alcoholic beverages (liquor, whiskey, vodka) |

**Table S1**. Comparison of metabolic parameters between women included and excluded

| Variables | Women included in the study (n=44) | | | Women excluded from the study (n=5) | | | p* | |  |
| --- | --- | --- | --- | --- | --- | --- | --- | --- | --- |
| Glucose | 109,66 | ± | 34,21 | 153,40 | ± | 110,69 | | 0,428 | |
| Insulin | 27,14 | ± | 34,21 | 28,12 | ± | 15,77 | | 0,884 | |
| HOMA-IR | 6,80 | ± | 3,39 | 9,64 | ± | 5,57 | | 0,105 | |
| HOMA-beta | 259,46 | ± | 169,95 | 238,82 | ± | 192,14 | | 0,800 | |
| HbA1c | 6,23 | ± | 1,06 | 6,80 | ± | 2,72 | | 0,666 | |
| TG (mg/dL) | 141,55 | ± | 57,28 | 193,40 | ± | 125,57 | | 0,411 | |
| TC (mg/dL) | 177,66 | ± | 31,76 | 197,20 | ± | 47,0 | | 0,220 | |
| HDL-c (mg/dL) | 47,43 | ± | 10,38 | 40,60 | ± | 4,72 | | 0,156 | |
| LDL-c (mg/dL) | 105,61 | ± | 28,21 | 124,60 | ± | 36,40 | | 0,172 | |
| VLDL-c (mg/dL) | 24,75 | ± | 7,20 | 31,80 | ± | 17,19 | | 0,413 | |
| PCR (mg/dL) | 1,22 | ± | 1,01 | 1,08 | ± | 1,11 | | 0,782 | |

HbA1c Glycosylated Hemoglobin (< 5,7%); TG: Triglycerides (<150 mg/dL); TC: Total cholesterol (<190 mg/dL); HDL-c: High-density lipoprotein (>40 mg/dL); LDL-c: Low-density lipoprotein (<130 mg/dL); VLDL-c: Very-low-density lipoprotein. Independent T-test. *p: ≤0,05

**Table S2**. Comparison of anthropometric parameters between women included and excluded

| Variables | Women included in the study (n=44) | | | Women excluded from the study (n=2) | | | p* | |  |
| --- | --- | --- | --- | --- | --- | --- | --- | --- | --- |
| Age (years) | 40,591 | ± | 8,75 | 44,50 | ± | 10,60 | | 0,542 | |
| Weight (kg) | 122,83 | ± | 18,14 | 110,27 | ± | 2,93 | | 0,338 | |
| Height (m) | 1,59 | ± | 0,06 | 1,55 | ± | 0,04 | | 0,356 | |
| BMI (kg/m²) | 48,60 | ± | 6,88 | 45,91 | ± | 1,29 | | 0,587 | |
| WC (cm) | 131,22 | ± | 12,61 | 130,87 | ± | 1,23 | | 0,970 | |
| HC (cm) | 145,64 | ± | 13,75 | 143,07 | ± | 0,10 | | 0,795 | |
| NC (cm) | 41,82 | ± | 3,13 | 37,65 | ± | 0,21 | | 0,069 | |

BMI, body mass index; WC, waist circumference (< 80 cm); HC, hip circumference; NC, neck circumference (≤ 34cm). Independent T-test. *p: ≤0,05
